# Exploring the antecedents of AI adoption for effective HRM practices in the Indian pharmaceutical sector

**DOI:** 10.3389/fphar.2023.1215706

**Published:** 2023-11-14

**Authors:** Manisha Goswami, Supriya Jain, Tabish Alam, Ahmed Farouk Deifalla, Adham E. Ragab, Rohit Khargotra

**Affiliations:** ^1^ Institute of Business Management, GLA University, Mathura, India; ^2^ CSIR-Central Building Research Institute, Roorkee, India; ^3^ Structure Engineering and Construction Management, Future University, New Cairo, Egypt; ^4^ Department of Industrial Engineering, College of Engineering, King Saud University, Riyadh, Saudi Arabia; ^5^ Institute of Materials Engineering, Faculty of Engineering, University of Pannonia, Veszprém, Hungary

**Keywords:** artificial intelligence adoption, human resource management effectiveness, organization preparedness, perceived benefits, technological readiness, competitive pressure, organizational culture

## Abstract

**Purpose:** The aim of this research is to investigate the factors that facilitate the adoption of artificial intelligence (AI) in order to establish effective human resource management (HRM) practices within the Indian pharmaceutical sector.

**Design/methodology/approach:** A model explaining the antecedents of AI adoption for building effective HRM practices in the Indian pharmaceutical sector is proposed in this study. The proposed model is based on task-technology fit theory. To test the model, a two-step procedure, known as partial least squares structural equational modeling (PLS-SEM), was used. To collect data, 160 HRM employees from pharmacy firms from pan India were approached. Only senior and specialized HRM positions were sought.

**Findings:** An examination of the relevant literature reveals factors such as how prepared an organization is, how people perceive the benefits, and how technological readiness influences AI adoption. As a result, HR systems may become more efficient. The PLS-SEM data support all the mediation hypothesized by proving both full and partial mediation, demonstrating the accuracy of the proposed model.

**Originality:** There has been little prior research on the topic; this study adds a great deal to our understanding of what motivates human resource departments to adopt AI in the pharmaceutical companies of India. Furthermore, AI-related recommendations are made available to HRM based on the results of a statistical analysis.

## Introduction

Human resource management functions have evolved manyfold in the past few decades from a pure administrative job (Combs et al., 2005) to a strategic HR job (Renkema et al., 2017), that is, from strategic HRM to technology integration in the HRM procedure (Dhamija and Bag, 2020), and now, artificial intelligence has transformed every facet of HRM (Rao and Verweij, 2017; Alghnimi et al., 2020). Key drivers for such evolution are the advancements in technology, which are taking place worldwide; as per the report (Aayog, 2018), global investment in AI has been increased with an annual rate of 50% (Pillai and Sivathanu, 2020).

Artificial intelligence (AI) is transforming the way human resource management (HRM) is being carried out across various industries. The Indian pharmaceutical sector is no exception to this trend. AI-powered HRM practices can enhance the efficiency of HR processes, improve the quality of talent acquisition and retention, and contribute to better decision-making (Muduli and Trivedi, 2020; Wheeler and Buckley, 2021). Several studies have highlighted the benefits of AI-powered HRM practices (Ahmad Salleh and Janczewski, 2018; Cruz-Jesus et al., 2018). In a study conducted by Deloitte, 38% of the companies in India are planning to invest in AI-powered HRM practices in the next 2 years (Deloitte, 2021). Furthermore, a study by the National Association of Software and Services Companies (NASSCOM) predicts that the adoption of AI-powered HRM practices will result in a 20%–30% reduction in HR-related costs (NASSCOM, 2021). However, according to a report by NASSCOM and Ernst & Young (EY), the Indian pharmaceutical industry has been slow in adopting AI and other emerging technologies. The report reveals that only 25% of Indian pharmaceutical companies are currently using AI and only 12% have implemented the Internet of Things (IoTs) technology (Ernst & Young, 2019), although the Indian pharmaceutical sector is one of the fastest growing industries globally and is expected to grow to $65 billion by 2024. However, the sector faces several challenges, including increasing competition, pricing pressure, and the need for innovation. Effective HRM practices are crucial for the sector to address these challenges and maintain its growth trajectory (Charlier and Kloppenburg, 2017).

Despite the potential benefits of AI-powered HRM practices, the adoption of AI in the Indian pharmaceutical sector is still at a nascent stage (Pillai et al., 2022). Furthermore, in the context of AI-powered HRM practices, the technology should be aligned with the HR tasks and functions required in the Indian pharmaceutical sector (Pandey, 2020; Mahmoud, 2021; Prikshat et al., 2023). This includes tasks such as talent acquisition, retention, and employee engagement. If the technology is well-suited to these tasks, it is more likely to be adopted by organizations in this sector. The task-technology fit theory also suggests that the adoption of technology is influenced by other factors, such as individual and organizational factors (Goodhue and Thompson, 1995; Lin, 2014; Alyoussef, 2023). This includes factors such as the perceived usefulness and ease of the use of the technology, organizational support, and employee resistance. These factors can either facilitate or hinder the adoption of AI-powered HRM practices in the Indian pharmaceutical sector.

Therefore, understanding the task-technology fit theory and its implications is crucial for organizations in the Indian pharmaceutical sector to effectively adopt AI-powered HRM practices. It can help organizations evaluate the fit between the technology and the HR tasks required in the sector, identify potential barriers to adoption, and design strategies to promote the adoption of AI-powered HRM practices. Moreover, this sector faces several challenges, such as a lack of awareness about AI technology, resistance from employees, and the cost of adoption (Li et al., 2023). Therefore, there is a need for empirical research to identify the factors that influence the adoption of AI-powered HRM practices in the sector.

Thus, this study aims to address this gap by exploring the antecedents of AI adoption for effective HRM practices in the Indian pharmaceutical sector. The study is expected to provide insights into the organizational, technological, and individual factors that influence the adoption of AI-powered HRM practices. The findings of this study will contribute to the existing literature on AI adoption in HRM practices and also provide valuable recommendations for organizations in the sector to effectively adopt AI-powered HRM practices and achieve better outcomes.

However, the Indian pharmaceutical industry operates under a complex and evolving regulatory environment (Imran et al., 2013). Introducing AI-based solutions requires compliance with strict guidelines from various regulatory bodies like the Central Drugs Standard Control Organization (CDSCO) and the Indian Council of Medical Research (ICMR). These bodies have been cautious in approving AI-driven technologies due to concerns about data privacy, ethical implications, and patient safety (Salalli et al., 2023).

Effective implementation of AI in the pharmaceutical industry relies on access to vast amounts of high-quality patient data (Blanco-González et al., 2023). However, data privacy and security concerns pose significant barriers. Indian patients are particularly sensitive to data sharing, and there is a lack of comprehensive data protection laws to address these concerns adequately. Furthermore, the adoption of new technologies, including AI, in the Indian pharmaceutical industry is influenced by cultural beliefs, practices, and socioeconomic factors. Physicians and healthcare professionals might be hesitant to trust AI-driven diagnoses or treatment recommendations, leading to slow adoption and resistance to change (Yokoi et al., 2021).

## Theoretical background and hypothesis development

The technology acceptance model (TAM) proposes that perceived usefulness and the perceived ease of use are the main determinants of the user acceptance of technology (Davis, 1985). However, this current study is based on the extension of the TAM model, that is, the task-technology fit (Howard and Hair Jr, 2023), as it provides a more comprehensive perspective by not only considering user perceptions and attitudes toward technology but also the fit between the technology and the tasks performed by the user. The task-technology fit (TTF) theory has been widely applied and tested in various contexts and industries, including the adoption of new technologies in business, healthcare, and education, and many other domains (Faqih and Jaradat, 2021). It has been useful in predicting user acceptance and adoption of technologies and in identifying potential barriers to adoption that can be addressed to facilitate a successful implementation (Abdekhoda et al., 2014; Taherdoost, 2022).

The main rationale of the TTF (technology acceptance model, later extended as TAM2 and TAM3) is to understand and predict users’ acceptance of new technology (Lai, 2017). Developed by Fred D. Davis in the 1980s, the TTF theory seeks to explain why individuals embrace or reject information technologies in various contexts (Davis, 1989).

In other words, TTF focuses on the fit between the characteristics of the technology and the requirements of the task or job (Dishaw and Strong, 1999). Therefore, TTF is a more suitable approach when the goal is to understand the role of technology in supporting specific tasks or processes in an organization. It provides a more nuanced view of how technology can be used to enhance task performance and can be a useful tool for organizations to ensure that the technology they adopt is a good fit for the tasks performed by their employees (Chang et al., 2023).

The TTF model has been widely researched to examine the adoption of technology in areas like healthcare, big data, banking and finance, and training management systems (Tam and Oliveira, 2016; Sharif et al., 2019). Furthermore, numerous studies are conducted on TOE, assessing the factors in successful AI implementation at the individual level in various domains (Alsheibani et al., 2020; Chen et al., 2021; Oyetunde et al., 2022). So, keeping this theory as the base, this study aims to identify the antecedents of AI adoption for effective HRM practices in the Indian pharmaceutical sector.

Overall, the TTF theory provides a structured framework for researchers and practitioners to assess users’ attitudes and behaviors toward technology adoption, helping organizations make informed decisions and strategies to enhance technology acceptance and successful implementation (Yen et al., 2010).

## Hypothesis development

**FIGURE 1:**
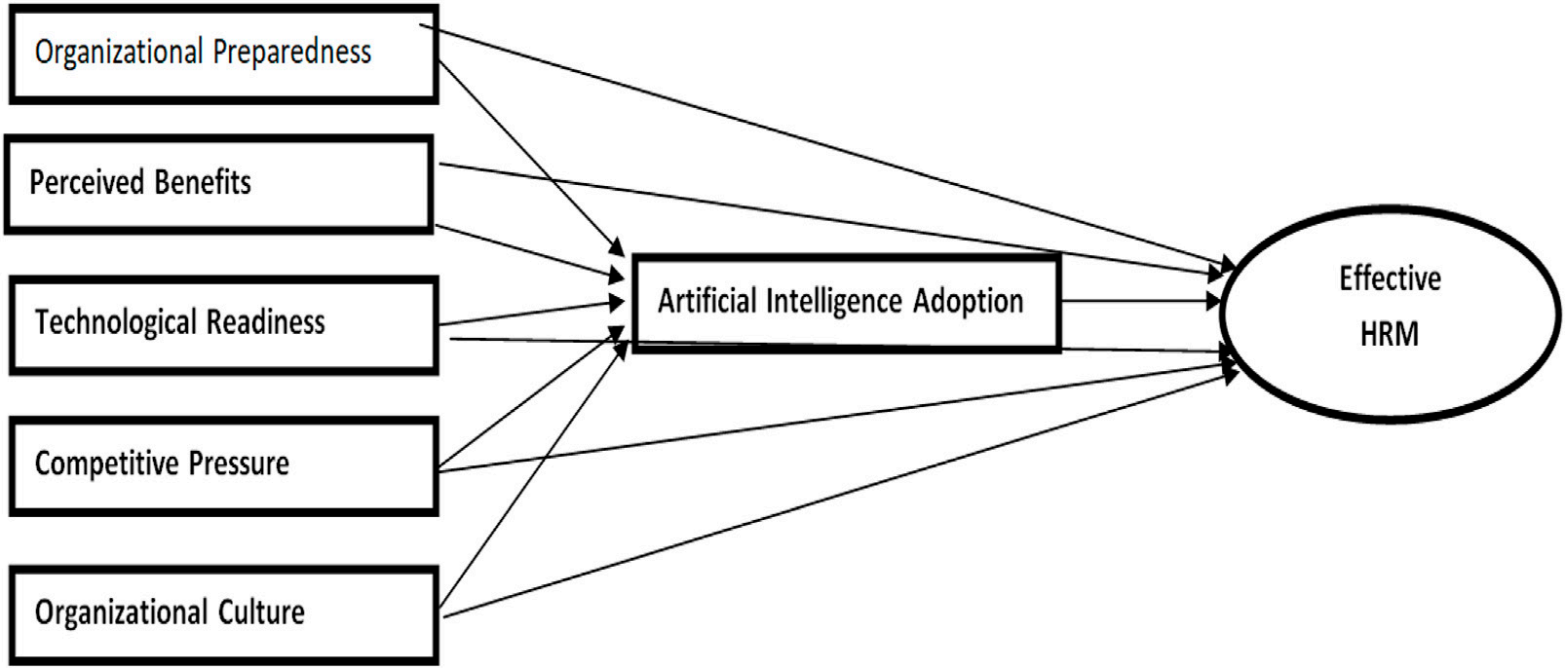
Conceptual framework.

Based on the literature review, we discuss the hypothetical relationship and conceptual framework shown in Figure 1.

### Artificial intelligence and effective HRM

Artificial intelligence will have an impact on human resources (Jia et al., 2018; Johansson and Herranen, 2019; Chowdhury et al., 2023). When the human element of human resources is integrated with the intelligence of technology, organizations will be able to offer better working circumstances to candidates and workers (Hashimoto et al., 2018). In addition, AI will help in completing the crucial task faster and better (Kim, 2020). AI-based HR solutions increase worker productivity (Oyetunde et al., 2022). It can analyze, forecast, diagnose, and develop into a more capable asset by focusing on employee requirements and outcomes (Toh et al., 2019). Businesses should implement AI technologies that align with their needs and corporate culture (Wamba et al., 2017). They should also create the necessary digital maps. Human resource management has evolved significantly as it has grown more contemporarily (Wijayati et al., 2022). This is due to the fact that digitization has spread to time-consuming activities within its many businesses (Baldegger et al., 2020). HRM has worked out how to employ previous technologies, such as the computer and the internet, to enhance market competition, cost-effectiveness, and the output (Agarwal et al., 2022). The McKinsey analysis estimates that AI will have a $13 trillion impact on the global economy (Brynjolfsson and Mcafee, 2017). Everyone believes that machine learning will affect all corporate functions, including human resources (Sahoo et al., 2022). Experts in human resources will employ technology to improve how individuals collaborate (Vrontis et al., 2022). By utilizing tested data, the industry can be enhanced and a more rational work atmosphere can be created (Chen, 2019; Jain et al., 2022; Goswami et al., 2023). Such a conducive environment will help in creating a positive workforce for the Indian pharmaceutical sector, which is the need of the hour after the COVID 19 outburst. Therefore, the following hypothesis can be formulated:

H1 Artificial intelligence adoption has a significant impact on effective HRM in the Indian pharmaceutical sector.

### Organizational preparedness

Organizational preparedness comprises organizational structures, processes, and other resources that promote the use of technology (Goodhue and Thompson, 1995; Yang et al., 2015; Wang and Siau, 2019; Jöhnk et al., 2021). According to the previous study, the characteristics such as a company’s size (Kaufman, 2015), the level of formalization, resource availability, and managerial support, all influence its readiness to adopt new technologies (Patel and Cardon, 2010). According to the resource-based view (RBV), an organization’s financial resources impede the adoption of AI in this manner (Tarannum and Farheen, 2020). Few studies on human resource systems suggest that the amount of knowledge in the HR department effects the adoption of new technologies (Stone et al., 2015).

AI use in the workplace is still in its early stages, and larger enterprises currently have more resources than smaller firms; thus, we believe that larger companies will employ more HRM-related AI solutions. AI can give businesses a competitive advantage, compelling them to use it (Benbya et al., 2020). An organization’s preparedness is an important factor in how new IT innovations are adopted and implemented (Alam et al., 2016; Hangl et al., 2022). Organizations that foster an open environment for innovation, demonstrate support for it, and provide adequate resources are more likely to embrace new IT innovations or engage in adoptive behaviors than their competitors (Wang et al., 2010; Wang and Siau, 2019), keeping in mind top management’s strategic direction, how they perceive and understand new AI HRM solutions, and how they feel about AI adoption in HRM. This is due to the fact that AI adoption significantly alters the traditional function of HRM (Chen, 2019).

Organizational preparedness to adopt AI must be tailored to the unique characteristics of each company, such as its industry or organizational structure (Jöhnk et al., 2021; Hradecky et al., 2022). Furthermore, because of how organizations evaluate themselves, the results of readiness models are frequently skewed (Fischer et al., 2020). However, before successfully adopting new technologies, organizations must be prepared and have the necessary capabilities (Daradkeh, 2019). An organization’s preparedness to adopt AI determines how well an organization is prepared to fully realize the promise of an innovation (Awa et al., 2016). Thus, the following can be hypothesized:

H2 Organizational preparedness has a significant impact on artificial intelligence adoption in the Indian pharmaceutical sector.

H3 Organizational preparedness has a significant impact on effective HRM in the Indian pharmaceutical sector.

### Perceived benefit

Perceived benefits are views about the benefits of a behavior in response to a real or perceived threat (Fischer et al., 2020). The perceived benefits of using AI are an important component of the research. People believe a technique is superior to other approaches because of its perceived benefits (Kambur and Akar, 2022). It has been discovered that AI’s perceived benefits diminish resistance to its adoption (Hradecky et al., 2022). HR managers are less likely to adopt AI if they are not sufficiently informed about its benefits and effectiveness (Guenole and Feinzig, 2018). Another important factor influencing attitudes is the expense of implementing AI (Jöhnk et al., 2021). The perceived benefits of adopting AI can be assessed based on its functionality, cost, and predicted benefits (Alam et al., 2016). As a result of the COVID pandemic, businesses have realized that automation and AI can help make work less personal (Gaur et al., 2021). As a result, how people perceive AI is determined by how it works, how much it costs, and what features it includes (Pillai and Sivathanu, 2020). All of these elements have an impact on how the decision-maker views the benefits of utilizing AI when more benefits are visible (Baig et al., 2019). Drawing on the aforementioned discussion, the following relations are hypothesized:

H4 Perceived benefits have a significant impact on artificial intelligence adoption in the Indian pharmaceutical sector.

H5 Perceived benefits have a significant impact on effective HRM in the Indian pharmaceutical sector.

### Technological readiness

AI adoption is primarily concerned with the qualities of the current technology and the ease with which an organization might embrace new technology (London, 2019; Sharma et al., 2022a). Technological readiness includes all of a company’s internal and external technologies, as well as their use in the business (Bhardwaj et al., 2020). The level of technological readiness and awareness of an organization’s employees are also major variables in the adoption of artificial intelligence. This is due to the fact that these factors influence the decision-making process and AI application. The HR personnel should also be aware of how AI fits into the overall business strategy (Pillai et al., 2022; Rahman et al., 2022). Technical experience makes it easier to embrace AI and integrate it with the existing HR systems; a company will not adopt a technology unless it has the essential knowledge and ability (Obeidat, 2016; Wang and Siau, 2019). Additionally, the successful use of AI has resulted in the reduction of numerous expenses, the effective and efficient use of resources, and the attainment of goals (Hedt and Pagano, 2011; Muduli and Trivedi, 2020). AI requires adaptive networks, software, hardware, and data deployments from IT (Wamba et al., 2017). According to research, organizations that have a strong technology infrastructure and the capacity to keep it current are better prepared for AI adoption (Jarrahi, 2018; Goswami, 2021). Furthermore, it has been suggested that a company’s technological preparedness is determined by factors other than its physical infrastructure. Drawing on the aforementioned discussion, the following relations are hypothesized:

H6 Technological readiness has a significant impact on artificial intelligence adoption in the Indian pharmaceutical sector.

H7 Technological readiness has a significant impact on effective HRM in the Indian pharmaceutical sector.

### Competitive pressure

The pressure that a company believes its competitors are putting on it is known as competitive pressure (van Esch et al., 2021). According to economic studies and surveys, as the global economy shifts toward a knowledge-based, free market economy, there is a greater competitive strain. Firms may feel compelled to adopt new IT innovations in order to remain competitive as a result of this pressure. Rizk (2020) asserted that due to intense competition, AI adoption may become a strategic necessity. Competitive pressure has a significant impact on the rate of AI adoption. Hossain et al. (2017) and Malik et al. (2021) discovered that pressure from trade partners and pressure from competitors are significant determinants of whether or not a corporation will use AI adoption. AI is still in its infancy in HRM; its apps are now affordable enough for use and have few technological issues. Drawing on the aforementioned discussion, the following relations are hypothesized:

H8 Competitive pressure has a significant impact on artificial intelligence adoption in the Indian pharmaceutical sector.

H9 Competitive pressure has a significant impact on effective HRM in the Indian pharmaceutical sector.

### Organizational culture

Organizational culture refers to how the workplace influences how employees behave, think, and feel about their jobs (Wilkens, 2020). Despite extensive research on organizational culture, no one can agree on a single definition. Despite the fact that there is no universally accepted definition of an organizational culture, it is commonly defined as “a collection of shared assumptions, values, and beliefs that are reflected in its practices and goals and help its members understand how the organization works”. Some believe that the organizational culture of a company is what keeps it together (Gaur et al., 2021; Wheeler and Buckley, 2021). An organization’s culture is a set of shared principles that distinguishes it from other organizations, which creates effective HRM practices. Something that all members of the group agree on is that the best strategy for maximizing the return on AI investments is to change organizational cultures (Cruz-Jesus et al., 2018). When AI is used correctly, organizations can build their cultures and that strong cultures can help firms use AI more effectively (Vrontis et al., 2022). AI and related technologies, such as machine learning, neural networks, and virtual assistants, have a significant impact on how businesses operate and are structured (Hangl et al., 2022). These excellent findings will undoubtedly persuade many businesses to invest in additional AI solutions. Drawing on the aforementioned discussion, the following relations are hypothesized:

H10 Organizational culture has a significant impact on artificial intelligence adoption in the Indian pharmaceutical sector.

H11 Organizational culture has a significant impact on effective HRM in the Indian pharmaceutical sector.

### Mediation hypothesis

This study delves into the mediating role of artificial intelligence on both independent and dependent variables. AI is widely recognized as a crucial growth driver that enhances business structures (Wamba et al., 2017; Kshetri, 2020; Kambur and Akar, 2022). Human intelligence involves the capacity to perceive, analyze, learn from past experiences, and devise innovative solutions to intricate problems (Mahmoud, 2021). In the Indian pharmaceutical sector, companies often face a substantial volume of job applications for limited positions. AI-powered chatbots have the potential to streamline and expedite the screening and filtering of applications, resulting in significant savings in time and effort for HR professionals. This enhanced efficiency and cost-effectiveness can lead to a perception of increased efficiency, which can ultimately contribute to more effective human resource management (Guenole and Feinzig, 2018). AI is vital in optimizing recruitment systems as it simplifies and reduces the cost of tasks, like application screening, while simultaneously enhancing the hiring quality (Muhaimin et al., 2020). Adopting AI in human resource operations can lead to greater efficiency and resource optimization (Sharma et al., 2022b). AI is perceived in human resource management as a means of expediting personnel hiring and selection processes while avoiding favoritism or nepotism (Reddy et al., 2019). This approach can facilitate staff development and improve their performance (Vinichenko et al., 2020). Drawing on the aforementioned discussion, the following relations are hypothesized:

H12 Adoption of artificial intelligence mediates the relationship between perceived benefits and effective HRM in the Indian pharmaceutical sector.

H13 Adoption of artificial intelligence mediates the relationship between competitive pressure and effective HRM in the Indian pharmaceutical sector.

H14 Adoption of artificial intelligence mediates the relationship between technological readiness and effective HRM in the Indian pharmaceutical sector.

H15 Adoption of artificial intelligence mediates the relationship between the organizational culture and effective HRM in the Indian pharmaceutical sector.

H16 Adoption of artificial intelligence mediates the relationship between organizational preparedness and effective HRM in the Indian pharmaceutical sector.

The present study aims to address the gaps in the literature by examining the influence of key factors, such as organizational preparedness, perceived benefits, technological readiness, competitive pressure, and organizational culture, on the adoption of artificial intelligence in the Indian pharmaceutical sector. A conceptual framework (Figure 1) has been developed to illustrate the relationships between the aforementioned variables and their impact on AI adoption, which, in turn, leads to effective human resource management. The study will employ partial least squares structural equation modeling (PLS-SEM) to analyze the proposed framework. The adoption of AI is expected to bring significant improvements to HRM in the Indian pharmaceutical sector.

## Methodology

This descriptive research focused on understanding the impact of organizational preparedness (OP), perceived benefits (PBs), technological readiness (TR), competitive pressure (CP), and organizational culture (OC) on effective HRM directly and via the mediation of artificial intelligence adoption in pharmaceutical companies in India.

An Indian pharmaceutical company’s employee working at a senior position, having an experience of 5 years and more in the sector, were considered as respondents for collecting primary data by the convenience sampling method (Vardarlier and Zafer, 2020). A self-administered structured questionnaire in English language with the Likert scale was used, following the 5-point Likert scale ranging from 1 (strongly disagree) to 5 (strongly agree); both of these helped in reducing response biases (Anjum et al., 2022; Rahman et al., 2022).

This study has used the scale adapted by the earlier researcher’s research work on building effective HRM practices via the adoption of artificial intelligence (Venkatesh and Davis, 1996). The four-item scale used in the study of OP has been adapted from the study of Hossain et al. (2017). PBs consist of the four-item scale adapted from Reddy et al. (2019); TR consists of the five-item scale adapted from Hedt and Pagano (2011) and Muduli and Trivedi (2020). The five-item scale of OC was adapted from the study of Wilkens (2020). Effective HRM AI has been adapted from the various authors’ research (Tambe et al., 2019). Competitive pressure was measured using the three-item scale adapted from the study of Gaur et al. (2021).

The content face validity was confirmed by two experts of the pharmaceutical sector. The suggestion provided by them was fully incorporated in the questionnaire, and for measuring the effectiveness of the questionnaire, a pilot study was conducted among pharmaceutical employees. The PLS-SEM statistical tool is used for analysis purpose, which even allows us to run the small sample size (Hossain et al., 2017; Malik et al., 2021); even non-normal distributed data can be analyzed using PLS-SEM (Hair et al., 2017a). This study circulated 250 questionnaires and received 150 responses; a response rate of 60% was recorded, which is within the threshold limit. The time period of the study was November 2022 to January 2023. To reduce the desirability biases, incomplete and incorrect responses were eliminated from the study, which, thus, left us with 88 responses for analysis purpose. Statistical and procedural methods were used to reduce the common method biases (Podsakoff et al., 2003). Additionally, when models contain a large number of constructs and items, PLS-SEM offers solutions for small sample sizes (Fornell and Larcker, 1981; Willaby et al., 2015; Hair et al., 2017b). A small sample size is ideal for PLS-SEM analysis since the population being studied is homogeneous (Cochran, 1977).

### Demographic profile

In the demographic profile of the 88 respondents of the pharmaceutical sector, out of which only eight were female individuals and the remaining 80 were male individuals, there is a dearth of female workforce in India. The respondents fall in the age bracket of 30–39 years old and have received responses from all parts of the country, namely, Vadodara, Ahmedabad, Baddi, Sikkim, Kolkata, Visakhapatnam, Hyderabad, Bangalore, Chennai, Mumbai, and Pune.

### Multicollinearity assessment

Using the variance inflation factor (VIF) to measure multicollinearity, the researchers found that none of the values in Table 1 were above the threshold of 5, indicating the absence of multicollinearity (Hair et al., 2017a).

**TABLE 1:** Construct reliability and validity.

Construct	Item	Item loading	VIF	Cronbach alpha	Composite reliability	AVE
Artificial intelligence (AI)	AI1	0.76	1.70	0.88	0.92	0.73
	AI2	0.91	3.14			
	AI3	0.89	2.76			
	AI5	0.86	2.52			
Competitive pressure (CP)	CP1	0.82	2.24	0.90	0.92	0.71
	CP2	0.91	3.27			
	CP3	0.82	2.22			
Effective HRM (EH)	EH1	0.84	1.83	0.92	0.94	0.77
	EH2	0.89	2.76			
	EH3	0.83	2.38			
	EH4	0.90	3.29			
	EH5	0.91	2.31			
Organizational culture (OC)	OC1	0.80	3.62	0.88	0.91	0.67
	OC2	0.84	3.74			
	OC3	0.82	2.04			
	OC4	0.84	2.51			
	OC5	0.79	2.32			
Organizational preparedness (OP)	OP1	0.91	2.39	0.91	0.93	0.74
	OP2	0.86	2.00			
	OP3	0.86	2.05			
	OP4	0.88	3.65			
Perceived benefits (PBs)	PB1	0.82	3.07	0.87	0.91	0.72
	PB2	0.84	3.33			
	PB3	0.83	3.39			
	PB4	0.88	1.84			
Technological readiness (TR)	TR1	0.89	2.10	0.93	0.94	0.77
	TR2	0.83	2.16			
	TR3	0.91	2.44			
	TR4	0.891	3.34			
	TR5	0.867	2.31			

### Measurement model assessment

The measurement model presents the validity and reliability of the construct. Four measurements are addressed in this study: 1. indicator loading, 2. internal consistency reliability, 3. convergent validity, and 4. discriminant validity (Risher and Hair, 2017).

### Indicator loading

PLS-SEM results were assessed for indicator loading in this study. Table 2 exhibits the details of loadings crossing the minimum threshold value of >0.70 ranging from 0.76 to 0.91, indicating that the construct has explained more than 70% of indicator variance, thus ensuring construct reliability (Hair et al., 2019).

**TABLE 2:** Discriminant validity (Fornell–Larcker criterion analysis).

	AI	CP	EH	OC	OP	PB	TR
Artificial intelligence (AI)	0.86						
Competitive pressure (CP)	0.62	0.84					
Effective HRM (EH)	0.78	0.57	0.88				
Organizational culture (OC)	0.77	0.64	0.72	0.82			
Organizational preparedness (OP)	0.67	0.68	0.71	0.73	0.86		
Perceived benefits (PBs)	0.66	0.55	0.64	0.67	0.57	0.85	
Technological readiness (TR)	0.68	0.75	0.74	0.76	0.70	0.57	0.88

### Internal consistency

Internal consistency should be presented through Cronbach's alpha and composite reliability (Hair et al., 2019). The value of Cronbach alpha ranges from 0.87 to 0.93, and composite reliability values are 0.91–0.94; both values are quite above the minimum threshold value of >0.70 (J. Hair and Alamer, 2022), thus establishing the internal consistency.

### Convergent validity

Convergent validity assessed the relationship among the similar and same construct, and the average variance extracted (AVE) to be reported; the minimum threshold value is 0.50 or higher (Hair et al., 2019). In this study, AVE values range from 0.67 to 0.77, which is quite above the acceptable limit, as shown in Table 1, thus establishing convergent validity.

### Discriminant validity

Discriminant validity explains the distinction among the constructs (Hair and Alamer, 2022). By applying Fornell–Larcker’s criterion, the AVE score of the construct should be less than the shared variance for the entire model construct; this study also depicts the same situation, as shown in Table 2. Thus, discriminant validity is established as per Fornell–Larcker’s criterion.

Furthermore, the heterotrait–monotrait ratio (HTMT), shown in Table 3, also confirms discriminant validity, where the values are less than 0.90 (Roemer et al., 2021; Hair and Alamer, 2022) of all the reflective constructs, indicating that there is no correlation among the constructs.

**TABLE 3:** Discriminant validity (heterotrait–monotrait ratio).

	AI	CP	EH	OC	OP	PB	TR
Artificial intelligence (AI)							
Competitive pressure (CP)	0.700						
Effective HRM (EH)	0.863	0.622					
Organizational culture (OC)	0.874	0.713	0.795				
Organizational preparedness (OP)	0.742	0.746	0.769	0.821			
Perceived benefits (PBs)	0.755	0.630	0.704	0.770	0.630		
Technological readiness (TR)	0.756	0.820	0.800	0.834	0.756	0.626	

### Assessment of the structural model

As per the techniques provided for structure model assessment (Risher and Hair, 2017), collinearity must be evaluated using VIF values of all sets of predictors. As shown in Table 4, all the values of the VIF are below the minimum acceptable value of 5 (Hair et al., 2019). Thus, collinearity is not a concern in this study.

**TABLE 4:** VIF values.

	AI	EH
Artificial intelligence (AI)		2.932
Competitive pressure (CP)	2.610	2.630
Effective HRM (EH)		
Organizational culture (OC)	3.419	3.880
Organizational preparedness (OP)	2.639	2.680
Perceived benefits (PBs)	1.924	2.059
Technological readiness (TR)	3.293	3.333

### Hypothesis testing

After establishing reliability and validity through the measurement model using PLS-SEM, the structural model is examined, which indicates the relationship between endogenous and exogenous variables. The path coefficients were studied by applying the bootstrapping process (two-tailed, at 5,000 samples, and 95% confidence level) (Hair et al., 2019).

The structural model helps in verifying all the projected hypotheses. The results are shown in Table 5, which reveals that artificial intelligence had a strong positive relationship with effective HRM (H_1_) (*b* = 0.40; *t* = 3.71; *p* = 0.00); hence, H_1_ is supported. However, the relationship between competitive pressure and artificial intelligence (H_2_) could not be established in the study (*b* = 0.08; *t* = 0.72; *p* = 0.47); hence, it was found to be insignificant. However, competitive pressure had a strong negative relationship with effective HRM (H_3_) (*b* = −0.20; *t* = −2.14; *p* = 0.03), which indicates that with the increase in competitive pressure, the effectiveness of HRM would decrease (Wang and Siau, 2019); hence, H_3_ is supported. The statistical analysis also confirms that organizational culture had a strong positive relationship with artificial intelligence (H_4_) (*b* = 0.40; *t* = 3.24; *p* = 0.00); thus, H_4_ is also supported. However, the relationship between organizational culture and effective HRM (H_5_) (*b* = −0.04; *t* = 0.34; *p* = 0.73) could not be established in this study; similarly, organizational preparedness had an impact on artificial intelligence (H_6_) (*b* = 0.12; *t* = 1.15; *p* = 0.25), which was found to be insignificant; thus, H_5_ and H_6_ are not supported. However, organizational preparedness proved to have a strong positive relationship with effective HRM (H_7_) (*b* = 0.26; *t* = 2.44; *p* = 0.01). Similarly, the perceived benefit has a strong positive relation with artificial intelligence (H_8_) (*b* = 0.21; *t* = 2.01; *p* = 0.04); thus, H_7_ and H_8_ are supported. However, perceived benefits fail to establish a relationship with effective HRM in the study (H_9_) (*b* = 0.15; *t* = 1.51; *p* = 0.13). Similarly, the relationship of technological readiness with artificial intelligence also could not be established (H_10_) (*b* = 0.12; *t* = 0.78; *p* = 0.44). However, the relationship between technological readiness and effective HRM (H_11_) (*b* = 0.39; *t* = 3.56; *p* = 0.00) was found to be significant; hence, H_11_ is supported.

**TABLE 5:** Hypothesis and path coefficient.

Hypothesis		Path coefficient	*t*-value	*p*-value	f^2^	CI		Decision
						LL	UL	
H_1_	AI ->EH	0.396	3.71	0.00	0.200	0.159	0.584	Supported
H_2_	CP ->AI	0.084	0.72	0.47	0.008	−0.134	0.323	Not supported
H_3_	CP ->EH	−0.201	2.14	0.03	0.058	−0.397	−0.032	Supported
H_4_	OC ->AI	0.397	3.24	0.00	0.135	0.145	0.619	Supported
H_5_	OC ->EH	−0.039	0.34	0.73	0.002	−0.242	0.211	Not supported
H_6_	OP ->AI	0.118	1.15	0.25	0.015	−0.100	0.306	Not supported
H_7_	OP ->EH	0.258	2.44	0.01	0.093	0.048	0.461	Supported
H_8_	PB ->AI	0.214	2.01	0.04	0.070	0.016	0.440	Supported
H_9_	PB ->EH	0.146	1.51	0.13	0.039	−0.031	0.348	Not supported
H_10_	TR ->AI	0.117	0.78	0.44	0.012	−0.185	0.400	Not supported
H_11_	TR ->EH	0.391	3.56	0.00	0.172	0.168	0.606	Supported

Regarding effect sizes (f^2^), values of 0.35, 0.15, and 0.02 are considered large, medium, and small for f^2^, as shown in Table 5 and Graph 1; the effect size (f^2^) of artificial intelligence is found to be an important variable in assessing the effectiveness of HRM in the Indian pharmaceutical sector. Second, organizational culture was close to a medium impact in assessing artificial intelligence adoption in the Indian pharmaceutical sector. Furthermore, technological readiness is also identified in this research as an important variable to assess the effectiveness of HRM in the Indian pharmaceutical sector. Furthermore, it is observed that at places where the hypothesis has insignificant *p*-values, the effect size f^2^ of that same hypothesis is revealed to be very small, even less than the threshold value of 0.02 (Cohen, 1988; Hair et al., 2019).

### Mediating effects

As per Hair et al. (2017b)’s recommendations, a mediation analysis was assessed using SmartPLS. The results reveal the effective mediation of artificial intelligence adoption in developing an effective HRM system in pharmaceutical companies. As shown in Table 6, **PB ->AI ->EH** perceived benefits have a significant relationship with effective HRM through the mediation of AI adoption (**H**_**12**_ **)** (*b* = 0.15; *t* = 3.37; *p* = 0.00), indicating full mediation as the perceived benefit was found to reject the direct relation with effective HRM (**H**_**9**_ **)** (Boon et al., 2011). Furthermore, **CP ->AI ->EH** competitive pressure has a significant relationship with effective HRM through the mediation of AI adoption (**H**_**13**_) (*b* = 0.12; *t* = 2.24; *p* = 0.02), indicating partial mediation, as competitive pressure was found to have a direct impact on effective HRM (**H**_**3**_) (Tan and Nasurdin, 2011). Similarly, **TR ->AI ->EH** technological readiness is also proved to have a significant relationship with effective HRM via the mediation of AI adoption (**H**_**14**_) (*b* = 0.10; *t* = 3.59; *p* = 0.00), indicating partial mediation, as technological readiness was found to have a direct impact on effective HRM (**H**_**11**_) (Esen and Erdogmus, 2014). **OC ->AI ->EH** organizational culture also proved to have significant relationship with effective HRM through the mediation of AI adoption (**H**_**15**_) (*b* = 0.16; *t* = 2.44; *p* = 0.01), indicating full mediation, as organizational culture was found to reject the direct relationship with effective HRM (**H**_**5**_ **)** (Al-Musadieq et al., 2018). Furthermore, this study proves another partial mediation of **OP ->AI ->EH** AI adoption on the relationship between organizational pressure and effective HRM (**H**_**16**_) (Hmoud, 2021) (*b* = 0.15; *t* = 3.98; *p* = 0.00) as organizational pressure is proven to have a direct impact on effective HRM (**H**_**7**_) (Agarwala, 2003).

**TABLE 6:** Indirect effect.

	Original sample (O)	Standard error	*t*-statistics (|O/STDEV|)	*p*-value	Decision
**PB ->AI ->EH**	0.15	0.33	3.37	**0.00**	**Supported**
**CP ->AI ->EH**	0.12	0.38	2.24	**0.02**	**Supported**
**TR ->AI ->EH**	0.10	0.30	3.59	**0.00**	**Supported**
**OC ->AI ->EH**	0.16	0.40	2.44	**0.01**	**Supported**
**OP ->AI ->EH**	0.15	0.27	3.98	**0.00**	**Supported**

The bold values represent that mediation is proved in all cases, whether it be partial or full mediation, depending upon the result of the direct relationship between the variables.

### Coefficient of determination

The coefficient of determination (*R* ^2^) values indicate the ability of the exogenous latent variable to predict the endogenous latent variables (Cohen, 1988); *R* ^2^ is 0.66 for artificial intelligence adoption and 0.73 for the effective HRM endogenous latent variable, indicating that the other five latent variables (like perceived benefits, competitive pressure, technological readiness, organizational culture, and organizational pressure) significantly explain 66% (*R* ^2^ = 0.66) of variance in artificial intelligence adoption. Similarly, the six other latent variables (like perceived benefits, competitive pressure, technological readiness, organizational culture, organizational pressure, and artificial intelligence) explain 73% (*R* ^2^ = 0.73) of variance in developing effective HRM.

## Discussion

This study aimed at identifying the impact of factors like organization preparedness, perceived benefits, technological readiness, competitive pressure, and organizational culture on the adoption of AI and effective HRM. Additionally, the mediation of AI adoption is also measured in the study of pharmaceutical companies in India. The finding of the study reveals that organizational culture and perceived benefits are positively associated with AI adoption with competitive pressure, organizational preparedness, and technological readiness as an exception, which means organizational culture and perceived benefits support the adoption of AI in pharmaceutical companies, as the COVID 19 pandemic has made them realize the importance of an AI-enabled healthcare sector (Reddy et al., 2019) to trace the requirement and control the flow of inventory. However, competitive pressure, organizational preparedness, and technological readiness could not establish relationships with AI adoption as the availability of resources and commitment is a must for bringing structural changes to the organization (Garrison et al., 2015); in addition to this, overall, the government spending on healthcare sector was just 1% of the GDP before the pandemic (Goswami, 2021) could also be the reason of the unavailability of the required resources for introducing AI in pharmaceutical companies. Furthermore, this study shows the positive relationship of organization preparedness, technological readiness, competitive pressure, and AI with effective HRM, with organizational culture and perceived benefits as exceptions. Strengthening the literature further as organizational preparedness, technological readiness, competitive pressure, and AI have proved to have significant relationships with effective HRM in previous research studies (Guenole and Feinzig, 2018). Furthermore, AI adoption was reported to have a full mediation impact on PB ->AI ->EH and OC ->AI ->EH, but partial mediation was also reported in the study, which impacted the relationship between CP ->AI ->EH, TR ->AI ->EH, and OP ->AI ->EH. This study helps in understanding the important role played by artificial intelligence as a mediator of the established relationship between dependent and independent variables (Choudhury et al., 2022); as seen in Table 5, H_2_, H_5_, H_6_, H_9_, and H_10_ are not supported directly from the perspective of the Indian pharmaceutical sector, but with the mediation of artificial intelligence, full mediation was shown to be established among the non-supported hypotheses.

Thus, these results indicate that artificial intelligence adoption plays a significant role to play in improving the effectiveness of HRM in pharmaceutical companies in India. However, several studies have proved the significance level of AI adoption in developing effective HRM, but exogenous variables considered in the current study have not been studied together in any previous research. Moreover, the current model of the study is applied in Indian pharmaceutical companies, which was not considered previously.

### Managerial implication of the study

Artificial intelligence has had a significant impact on HRM practices, but little research has been conducted to determine how well it works within organizations (Wamba et al., 2017). Several HR experts discussed how AI works and why it is important to use it in hiring. Businesses must carefully consider what AI can do for them and why it should be incorporated into their hiring processes.

AI, in my opinion, can improve the accuracy and effectiveness of the hiring process, but it all depends on the organization. Each organization will have unique efficiency goals and AI requirements. If organizations are to fully benefit from using AI in recruitment, they must have access to it and enough time to put it to use. This is because many organizations, whether they realize it or not, believe they want to deploy AI. They may not require this level of efficiency or quality or they may lack the technical expertise to implement it.

Long-term organizational changes should be prioritized, and we should work to develop methods for teaching both humans and machines to recognize employee skills that will benefit the company in the long run. Recruiters used to perform repetitive tasks manually, but AI would make some of these tasks obsolete (Pillai et al., 2022). Recruiters can now delegate these mundane tasks to AI systems, freeing up their time and resources for more strategic issues (Pillai and Sivathanu, 2020).

AI assists recruiters in completing less time-consuming tasks in order to conduct direct applicant interviews. Application screening saves time during recruitment and AI can assist recruiters in identifying top candidates by generating short lists. When artificial intelligence is used in hiring, recruiters can access a larger prospect pool while doing less administrative work (Vardarlier and Zafer, 2020). One of the most amazing aspects of AI-based recruiting is that it allows recruiters to spend more time with the best talent management candidates (van Esch et al., 2021).

This study provides corporate executives in the pharmaceutical sector and HR managers with recommendations for improving the effectiveness of their HR departments. HR leaders and upper management should be aware that integrating AI into the HR system may have benefits that extend beyond the HR department and to the entire company. Before implementing artificial intelligence, organizations must be prepared, understand the benefits, and have the necessary technical skills. HR managers and leaders should conduct research before developing a strategy for integrating AI into the current system. The first step can be to determine which tasks, procedures, and processes need to be modified. People may consider whether tasks can be automated in order to increase their productivity and efficiency at work. Furthermore, the value that people contribute can be quantified, considering how people analytics can help you improve your individual performance, education, and career development, as well as your pay and benefits. However, it is critical to determine whether a major overhaul is required. Once this preliminary research is completed, a strategy for automating AI use can be developed. AI will be used more and more in future HR departments, particularly when communicating with applicants and assisting them in preparing for interviews.

### Limitations and scope for future research

This study provides important and novel information on the driving forces behind the use of AI in the HR department of pharmaceutical companies of India. However, the study has several limitations that must be addressed. First, we only looked at data from major cities. Second, we only considered the pharmaceutical industry as a whole. Third, we regard AI as a high-tech tool. However, AI’s cognitive capabilities differ significantly from those of traditional e-HRM tools. Many people believe that AI is more than just a technology (van Esch et al., 2021; Vrontis et al., 2022). Future research should look into people’s reactions to the idea of artificial intelligence technology replacing humans. AI can be used in a variety of ways by different cultures and nations. Because our study was limited to India, the current findings cannot be applied to other countries. Only 160 human resource managers from Indian pharmaceutical companies were involved. People believe that in the future, researchers should conduct comparable studies in other fields to learn more. Although the questions and ideas used in this study came from the studies conducted in Western countries, the survey was conducted in accordance with the rules to ensure high-quality data. It is suggested that India should develop its own scales for future investigations.

## Conclusion

Artificial intelligence-based technologies are increasingly being used in industries such as education, healthcare, and the environment. AI-powered solutions enable people to make decisions based on how they interact with their surroundings. As a result, the factors influencing AI adoption must be comprehended. AI shows potential to significantly alter the workplace and provide businesses with a competitive advantage (Tambe et al., 2019). As a result, practitioners and academics must understand what hinders AI implementation and what facilitates it. The rationale for the HR department’s adoption of AI will be examined in this paper. A thorough review of the literature was conducted to determine what factors might make it easier for people to accept new technologies. According to the research, organizational culture and how people perceive the benefits aid in the adoption of AI by pharmaceutical companies. All hypothesized relationships are confirmed by data analysis, proving the validity of the proposed model. The findings of the study should persuade strategic HR leaders in pharmaceutical companies of the benefits of using AI to build effective HR systems. AI-based HRM systems excel at many tasks, including hiring candidates faster, spending less money, and making the best use of available resources. AI shows potential to expand the number and diversity of hiring metrics. AI will become a critical tool for achieving HRM goals, such as hiring top talent, retaining them for longer periods of time, and developing their leadership potential.

## Data Availability

The raw data supporting the conclusion of this article will be made available by the authors, without undue reservation.
